# Editorial: Break the mental health stigma: mental health in the workplace

**DOI:** 10.3389/fpsyt.2024.1427097

**Published:** 2024-05-22

**Authors:** Eduardo Fernández-Jiménez, Daniela Acquadro Maran

**Affiliations:** ^1^ Department of Psychiatry, Clinical Psychology and Mental Health, La Paz University Hospital, Madrid, Spain; ^2^ Hospital La Paz Institute for Health Research (IdiPAZ), Madrid, Spain; ^3^ Faculty of Social Sciences and Communication, Universidad Europea de Madrid, Madrid, Spain; ^4^ Dep​artment​ of Psychology, University of Turin, Turin, Italy

**Keywords:** mental health, stigma, discrimination, workplace, mental health stigma

The workplace today is an environment where mental health issues are increasingly becoming a focus of interest and concern. However, in some organizations, there is still a stigma attached to this issue. This can lead to discriminatory actions and attitudes towards employees who live with mental disorders, both at the personal and public levels. In particular, the stigmatization of people living with mental disorders in the workplace has a number of detrimental effects such as emotional distress (feeling uncomfortable and embarrassed), lower self-esteem, difficulties in social relationships, and a lower likelihood of initiating or maintaining a psychological treatment. It can also lead to difficulties in workplace relationships and in getting support or other job benefits (1). This Research Topic aims to integrate twelve studies in order to pave the way towards breaking down the barriers of mental health stigma in the workplace. This Editorial encompasses pioneering research endeavors, from observational studies, validation studies of new assessment tools until intervention studies, and integrates the complexities of mental health in professional settings, offering insights, and solutions to foster supportive environments where individuals can thrive.

The COVID-19 pandemic has brought mental health challenges to the forefront, exacerbating previous problems and highlighting the need for comprehensive support systems. In this sense, the study by Xiong et al. shed light on the significant impact of the COVID-19 pandemic on the mental well-being among healthcare workers. From an observational design, this study provides valuable insights about the relationship between workplace factors, burnout, wellbeing, and sleep quality, highlighting the urgent need for targeted interventions to support frontline workers. In the same way, the systematic review and meta-analysis by Navinés et al. shows a substantial prevalence of burnout among medical residents during the first wave of the COVID-19 pandemic.

Furthermore, the COVID-19 pandemic has underlined the relevant role of resilience in order to cope with challenging workplaces. In this sense, Li et al. examines qualitatively the views of Chinese nurses on the factors (professional, practical, and personal) that contribute to resilience in the workplace. Additionally, addressing mental health stigma also requires a self-critical perspective on the part of health systems, as demonstrated by studies such as the one conducted by Li et al. More specifically, this national survey sheds light on the stigmatizing attitudes towards people living with mental disorders by non-mental health nurses from general hospitals in China. Therefore, this work emphasizes the need for widespread education and social awareness to combat stigma within healthcare settings.

Moreover, the study carried out by Cheng et al. explores the complex dynamics of workplace culture and its impact on employee engagement. By examining the influence of negative workplace gossip on work engagement through the moderating role of mindfulness and superior trust, this study underscores the importance of promoting supportive and positive workplaces, avoiding dysfunctional interpersonal patterns. Additionally, the relationship between leadership profile and mental health outcomes is a key area of study. For instance, Jalil et al. explore the mediating role of meaningful work in the relationship between empowering leadership and mental health outcomes among employees from small and medium enterprises in Malaysia. This research provides valuable insights into the factors that contribute to employee well-being and organizational success.

As organizations opt for taking care of employees’ mental health in the workplace, new and pioneering approaches to meet the multiple needs of workers are warranted. To illustrate, West at al. propose the integration of behavioral science and artificial intelligence to offer personalized mental health support. This pilot study demonstrates the potential of technology-driven solutions to promote access to mental health resources and foster wellbeing among employees. In the same way, Woodard et al. present a population health approach to address mental health issues in the workplace. To this end, they proactively provide regular mental health assessments of employees and, if they exceed the “at risk” threshold, a Care Concierge is offered to connect users with various resources.

Additionally, the systematic review conducted by Guo et al. highlights the psychological toll faced by interpreters in emotionally challenging situations (police interviews before domestic violence cases). This study underlines the importance of comprehensive support systems for professionals exposed to traumatic experiences in the workplace. Furthermore, Diaz et al. analyze the factors influencing the wish to die among French physicians. Results of this study show the relationship between burnout, work-related difficulties, personality traits (emotional stability, extraversion, and agreeableness) and an increased wish to die among physicians. Therefore, this study underscores the importance of addressing workplace stressors and promoting mental health support systems to mitigate the risk of suicidal ideation among healthcare professionals.

Finally, in addition to the immediate challenges posed by the COVID-19 pandemic, mental health stigma continues to be present over workplaces worldwide. Matousian et al. propose the development of a new instrument for quantifying and understanding mental health stigma in professional settings, specifically within the return-to-work process. Likewise, ensuring culturally sensitive approaches to mental health assessment is crucial for addressing the diverse needs of communities worldwide. In this sense, Odero et al. aimed to psychometrically validate two widely used screening tools for depression and anxiety (PHQ–9 and GAD–7) among nurses/midwives and Community Health Volunteers in Kenya.

In conclusion, the collective insights from the twelve contributions of this Research Topic highlight the multifaceted nature of mental health stigma in the workplace. Addressing this stigma warrants a comprehensive approach that encompasses a nuanced understanding of workplace dynamics, societal awareness, supportive leadership, as well as new assessment tools and innovative interventions. By integrating this knowledge into practical and actionable strategies, organizations can pave the way towards creating inclusive and supportive work environments that also care for the mental health of their employees.

## Author contributions

EF: Conceptualization, Supervision, Validation, Writing – original draft, Writing – review & editing. DA: Supervision, Validation, Writing – original draft, Writing – review & editing.
